# Instant messaging – one solution to doctor–student communication?

**DOI:** 10.3402/meo.v20.30593

**Published:** 2015-12-23

**Authors:** Ibtesham Tausif Hossain, Umair Mughal, Bashar Atalla, Mustafa Franka, Sarim Siddiqui, Mohammed Muntasir

**Affiliations:** School of Medicine, Imperial College London, London, UK

In the last decade, social media has infiltrated into many dimensions of life forming the fabric of communication and innovation. Instant messaging services such as WhatsApp Messenger (WhatsApp, Inc., California) have grown in popularity, with over 800 million monthly active users, making it the most popular instant messaging application worldwide (1). While this kind of technology has been embraced by a variety of professions to increase communication between colleagues, the communication systems within a hospital environment has remained quite stagnant, reminiscent of the period before smartphone and social media (2, 3).

Many hospitals still use outdated pager systems as the foundation for clinical communication. This is despite problems including long waiting times for the return of a page, high costs, frequent interruptions, and the inability to identify the location or identity of the caller (4). From a medical student's perspective, barriers to communication with supervisors present unique obstacles to learning. Clinical clerkship is largely unstructured with topics determined by presenting conditions of patients at the bedside and interests of the teacher. This has contributed to little consistency in delivery of the core undergraduate syllabus (5). It is further compounded by the fact that this model of opportunistic teaching naturally relies heavily on communication between students and teachers, and currently there is little in the way of an effective system in place to foster a conducive and productive experience.

However, the use of WhatsApp can provide a solution to this by providing subtle structure to an erratic environment. There is already evidence to suggest that students are willing to use instant messaging for academic purposes (6) highlighting a shift in perception who use these platforms primarily for social purposes (7).

The advantages of instant messaging between a group of students and their supervisor include rapid arrangement of *ad hoc* teaching, the identification of interesting patients, and a platform to discuss cases and ask for additional support during the less busy hours. One of the main benefits of this technology is the positive effect on student relations with supervisors. Instant messaging can serve to build student–doctor and student–student relationship in a controlled environment (8). Having undergone a clinical attachment in Obstetrics & Gynaecology at St Marys Hospital (London, UK), we as medical students analysed this method of communication and found it advantageous as it diminished hierarchy and was conducive to an enjoyable learning experience. The effectiveness has also been cited by a recent study carried out by Willemse (9) on a sample of student nurses.

The disadvantages of using an instant messaging service within a healthcare environment are upholding patient confidentiality and safety. However, if an effective protocol is put in place and anonymity is upheld, these issues can be easily overcome (3). These limitations therefore should not restrict its use in a formal capacity, but rather the pedagogy of social media and instant messaging should be used as a tool to foster professionalism in a digital world. In addition, applications such as *Seratis* and *Cureatr* (10), which employ the use of encrypted messaging, are currently under trial for their propriety in the healthcare environment. Educators face challenges in adapting new technologies, but they also have opportunities for innovation.

The scope of instant messaging is well known but has yet to penetrate the healthcare environment formally. Capitalising on this widely used technology can aid in the communication and ultimately learning experience of medical students. The wider potential of such technology is exciting and can provide an alternative mode of clinical communication between surgical and medical teams with the possibility of a purpose-built instant messaging service for healthcare professionals going beyond the advantages of medical education.

*Ibtesham Tausif Hossain* School of Medicine Imperial College London London, UK ibtesham.hossain11@imperial.ac.uk *Umair Mughal* School of Medicine Imperial College London London, UK *Bashar Atalla* School of Medicine Imperial College London London, UK *Mustafa Franka* School of Medicine Imperial College London London, UK *Sarim Siddiqui* School of Medicine Imperial College London London, UK *Mohammed Muntasir* School of Medicine Imperial College London London, UK 
